# Clarity of task difficulty moderates the impact of the explicit achievement motive on physical effort in hand grip tasks

**DOI:** 10.1371/journal.pone.0252713

**Published:** 2021-06-04

**Authors:** Michael Richter, Florence Mazeres, Kerstin Brinkmann

**Affiliations:** 1 Effort Lab, School of Psychology, Liverpool John Moores University, Liverpool, United Kingdom; 2 Geneva Motivation Lab, University of Geneva, Geneva, Switzerland; Aalborg University, DENMARK

## Abstract

Stable personality dispositions, like motives, are often assumed to exert a direct, stable impact on behavior. This also applies to the explicit achievement motive, which is supposed to influence the behavior that individuals select and how strongly they engage in it. Drawing on motivational intensity theory, we demonstrated in two studies that explicit achievement motive strength only predicted exerted force in a hand grip task if task difficulty was unclear. If task difficulty was clear, explicit achievement motive strength did not influence exerted force. Our findings suggest that the availability of information about the difficulty of motive satisfaction moderates the impact of the explicit achievement motive on behavior.

## Introduction

One of the main reasons for the interest in personality traits is the promise that they have stable links with behavior and consequently allow the prediction of behavior [1, 2]. This also applies to motives, a complementary personality construct that refers to the disposition—originating from childhood learning experiences—to strive for certain types of incentives[3–5]. All three main classes of motives that have been examined in the McClelland tradition [4], the achievement motive, the affiliation motive, and the power motive, lead individuals to engage in motive-relevant tasks. For instance, individuals with a strong achievement motive are considered to experience meeting standards of excellence as rewarding and therefore to seek to attain and exceed such standards [6]. Consequently, a strong achievement motive is postulated to drive individuals to actively select tasks that are instrumental for motive satisfaction (i.e., tasks that allow them to attain excellence). Moreover, it should make them willing to invest more effort and to be more persistent in such tasks [4, 7, 8]. Like research on the predictive power of personality traits, the literature on motives has strongly focused on behavior initiation and performance (i.e., how well individuals perform a task) [9, 10]. Other qualities of behavior, like effort (i.e., the investment of resources to perform a task) or disengagement, have been less frequently examined. The aim of this article is to extend the existing literature on motives by providing a theoretical analysis of the motive-effort relationship based on motivational intensity theory [11], and to provide a test of this framework by examining the effect of the explicit achievement motive on effort under conditions of clear and unclear task difficulty.

Motivational intensity theory suggests that two variables determine the amount of effort that one invests in tasks that are instrumental for attaining personal goals: task difficulty and success importance (i.e., the importance of successfully performing the instrumental task). Two additional variables, clarity of task difficulty and type of difficulty standard (fixed vs. unfixed) are predicted to determine how task difficulty and success importance influence effort. If task difficulty is clear and fixed, effort should be a function of task difficulty (the higher the difficulty, the higher the effort) up to a point where the required effort is no longer justified by the importance of succeeding in the task. If the required effort exceeds the amount of maximally justified effort, individuals should disengage and invest no effort. The underlying assumption is that individuals aim to avoid wasting resources that may be important for survival [11, 12]. Using task difficulty as an indicator of what is required allows individuals to attain this goal. If the difficulty of a task is not clear (i.e., unclear or unpredictable) or not fixed (i.e., the performer is free to perform the task at any difficulty level), task difficulty cannot be used as an indicator of what is required in these situations. Consequently, individuals use the next best indicator that allows them to minimize the waste of resources, and success importance should directly determine effort: the higher the success importance, the higher the effort [11–13]. Success importance will not allow individuals to invest exactly what is required, but it will allow them to avoid investing more than justified [12]. Motivational intensity theory can be used to derive predictions for other variables by identifying the theory’s components that should be affected by these variables [14]. For instance, researchers demonstrated that ability [15], gender [16], and pain [17] exert their impact on effort via influencing perceived task difficulty. Need for closure [18], grit [19], and ego involvement [20] influence effort via changes in perceived success importance. Some variables, like mood, implicit affect, and dysphoria, can affect both task demand and success importance [21–24].

**Fig 1:**
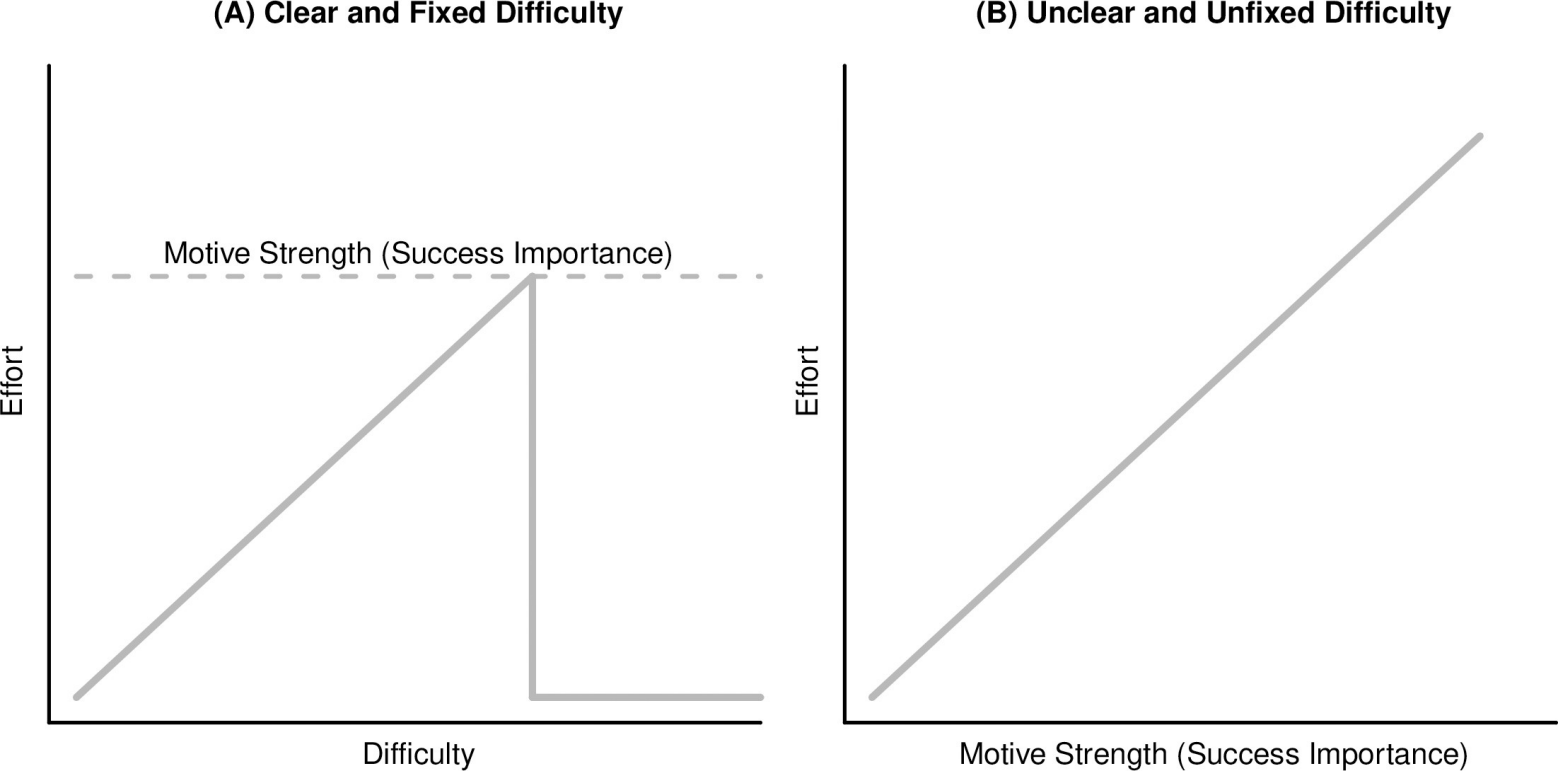
Theoretical predictions for the impact of the explicit achievement motive on effort.

Given that motives refer to how important it is for individuals to attain specific types of incentives, motives should influence effort by changing perceived success importance. In the case of the achievement motive, individuals with a strong motive should be more interested in succeeding in tasks that allow them to attain and demonstrate excellence (i.e., achievement tasks) than individuals with a weak motive. The strength of the achievement motive should thus determine how important success is in achievement tasks and how much effort one is willing to maximally invest in such tasks. In tasks with clear and fixed difficulty, motive strength should consequently not exert a direct impact on effort but limit the difficulty-effort relationship by determining the point at which individuals consider the required effort no longer to be justified and disengage. In task with unclear or unfixed task difficulty, motive strength should directly determine how much effort individuals invest in the task. The clarity of task difficulty and the type of difficulty standard should thus determine whether motives exert a direct or indirect influence on effort investment. Fig 1 displays these predictions for the impact of motive strength on effort.

It is important to note that the popular distinction between implicit and explicit motives [8, 25] does not translate into different predictions for implicit and explicit achievement motives regarding their effects on effort. Given that both subclasses of the achievement motive refer to the striving to attain and exceed standards of excellence, they should both exert their impact on effort by increasing the importance of succeeding in tasks that promise motive satisfaction. Implicit and explicit motives differ in many aspects [8, 25], like accessibility, specific behavioral consequences, or activating situational characteristics, but they should not differ regarding the mechanism by which they exert their impact on effort. For instance, the implicit achievement motive (need for achievement, nAch) refers to the nonconscious disposition to strive for excellence whereas the explicit achievement motive (self-attributed need for achievement, sanAch) refers to the self-attributed disposition of pursuing standards of excellence. Correspondingly, the explicit achievement motive can be assessed via self-reports, but the implicit motive needs to be measured using projective, implicit measures. Implicit motives are more predictive of spontaneous and non-declarative processes whereas explicit motives are better predictors of deliberate and declarative processes [25–27]. The implicit achievement motive is activated by tasks that offer an opportunity to improve one’s performance, whereas explicit, external challenges to demonstrate one’s excellence and enable social comparison activate the explicit achievement motive [8]. Explicit and implicit achievement motives may thus become activated in different types of situations, but both should exert their impact on effort by increasing the perceived success importance of tasks that are instrumental for motive satisfaction. The observable effects of explicit and implicit achievement motives may differ depending on the specific context, but their impact on effort should always be mediated by the same mechanism: changes in perceived success importance.

There is already research relevant to our analysis of the impact of the achievement motive on effort. The most direct evidence comes from a recent study by Mazeres and colleagues [28] in which they examined the influence of the implicit achievement motive on effort under conditions of clear and fixed task difficulty. In line with our theoretical analysis, the strength of the achievement motive had an indirect impact on effort under these conditions: Effort increased with increasing task difficulty in the strong-achievement-motive group but was low and independent of task difficulty in the weak-achievement-motive group. Brunstein and Schmitt [7] described a similar interaction between the implicit achievement motive and task difficulty. Individuals with a strong implicit achievement motive invested more effort in a moderately difficult task than in an easy or a very difficult task, whereas individuals with a weak implicit achievement motive invested low effort independent of task difficulty. Capa and colleagues [29, 30] found similar results examining the impact of the explicit achievement motive on effort. Individuals with a large positive difference between the motive to achieve success and the motive to avoid failure increased their effort investment as a function of increasing task demand. This adaption to task demand was not observed in individuals with a large negative difference between the two achievement motive components. In an applied context, Wegner and Schüler [31] showed that the implicit achievement motive predicted the effort invested in playing racquet games, which can be considered tasks with an unfixed task difficulty given that the difficulty level of each individual stroke is to some extent under the control of the individual.

There are thus already a few studies that revealed achievement motive effects congruent with our theoretical analysis. However, most of these studies focused on the interaction between the achievement motive and task difficulty under conditions of clear and fixed task difficulty. Empirical research has not yet examined the prediction that clarity of task difficulty determines the impact that the achievement motive has on effort. The presented studies aimed to close this gap by examining the impact of the strength of the explicit achievement motive on physical effort invested in hand grip tasks with clear and unclear difficulty. Following our theoretical analysis presented in the preceding paragraphs, we expected effort (i.e., the force exerted in the hand grip task) to be a direct function of the explicit achievement motive if participants worked on a task with an unclear difficulty. If participants worked on a task with a clear difficulty, we expected no relationship between the strength of the explicit achievement motive and effort. These hypotheses were not explicitly pre-registered but all predictions, methods, and analyses had been described in the Swiss National Science Foundation grant 100019_162390 before data collection started. All collected data are permanently and openly accessible at https://doi.org/10.24377/LJMU.d.00000077.

## Material and methods

### Participants and design

All presented studies have been conducted with the formal approval (approval number 17/NSP/055) of the ethic review committee (UREC) of Liverpool John Moores University (Liverpool, UK). All participants provided written consent. Sample size of Study 1 was determined in an a priori power analysis using G*Power [32] (alpha = .05, power = .80). Drawing on preceding work on achievement motive effects in physical tasks [31, 33, 34], we assumed an effect size of ρ = .40. We used the same sample size for Study 2, which led to a low power of .34 for the comparison of the correlation coefficients between the two difficulty conditions. All participants were recruited on the campus of a UK University. In Study 1, 40 participants performed a hand grip task with an unclear task difficulty and completed the achievement (sanAch), affiliation (sanAff), and dominance/power scales (sanPow) of the Personality Research Form (PRF) [35]. Due to a programming error, gender and age data of these participants were not recorded in this study. In Study 2, 40 participants (*M*_age_ = 27.75, *SD* = 14.94 for the entire sample, *M*_age_ = 26.10, *SD* = 15.24 in the clear-difficulty condition, *M*_age_ = 29.40, *SD* = 14.83 in the unclear-difficulty condition) performed a hand grip task with either an unclear task difficulty or a clear, easy difficulty standard before completing the achievement, affiliation, and power PRF scales. Allocation to the two difficulty standard conditions (unclear vs. clear) was randomized with the constraint that there had to be 20 participants in each condition. There were three male and 17 female participants in the clear-difficulty condition and eight male and 12 female participants in the unclear-difficulty condition. Both studies include three main elements: maximum force trials, the main hand grip task, and the PRF achievement, affiliation, and power scales.

### Tasks and questionnaires

#### Maximum force trials

In the maximum force trials participants were asked to squeeze a hand dynamometer (Vernier, Beaverton, OR) as hard as possible during a two-second measurement period. During the measurement period, the force that participants were exerting was presented on-screen. Moreover, after the measurement period, the maximum force exerted during the preceding measurement period was presented on-screen (“The maximum force that you exerted in this trial was XXX Newton”). During all maximum force trials, the experimenter, who was hired and unaware of the hypotheses, was in the same room as the participants and encouraged them to squeeze has hard as they could by asking them to exceed their maximum force of the preceding trial. Participants performed three maximum force trials before the main hand grip task and three maximum force trials after it. The main purpose of the assessment of participants’ maximum force was to control for interindividual differences in hand grip strength (e.g., gender-related difference [36]).

#### Hand grip task

The hand grip task followed a paradigm successfully employed in previous studies on motivational intensity theory [37, 38]. In the hand grip task, participants were asked to imagine that the dynamometer represented a clogged Ketchup bottle that they could free by exerting a force equal to or higher than a force standard (i.e., a specific force in Newton) determined by the computer. To support this cover story, a black-and-white drawing of a hand holding a reversed Ketchup bottle was presented on-screen throughout the task and a black-and-white drawing of a hand holding a bottle ejecting Ketchup replaced the default image at the end of successful trials. The task consisted of 60 trials each one starting with a four-second countdown followed by a two-second measurement period and a three-second feedback period. The force that participants were exerting on the dynamometer during the measurement period was measured and compared to the trial’s force standard. If the peak (maximum) exerted force was equal to or greater than the standard, the trial counted as success and the positive feedback drawing was displayed during the feedback period. If a participant’s peak (maximum) exerted force was less than the standard, the trial counted as failure and the presented drawing did not change (i.e., the default drawing of a hand holding a reversed Ketchup bottle remained on the screen). The force exerted in this isometric hand grip task is proportional to the amount of energy (adenosine triphosphate) invested for task success and provides thus a valid measure to test effort-related predictions in instrumental tasks [37].

To create a task version with unclear, unpredictable difficulty (Study 1 and Study 2), force standards were varied from trial to trial. Thirty different force standards were presented twice in random order to create the 60 task trials. To ensure that each participant would receive roughly the same number of possible and impossible trials, the list of 30 force standards was determined individually for each participant using participants’ maximum force assessed in the first three maximum force trials. If a participant’s maximum force exceeded 145 Newton (N), force standards were created by subtracting 120 N from the participant’s maximum force and increasing the force standard in a stepwise manner by adding 5 N. This resulted in 25 force standards equal to or below a participant’s maximum force and five force standards above the participant’s maximum force. For instance, if a participant’s pre-task maximum force was 181 N, the used force standards were 61 N, 66 N, 71 N,. . ., up to 206 N. If a participant’s maximum force did not exceed 145 N (one participant in Study 1, six participants in Study 2), 30 force standards ranging from 25 N to 170 N were used. To create the task version with clear difficulty (Study 2), the same easy force standard of 60 N (corresponding to the strength of a weak, low-effort handshake [39, 40]) was used in all trials and for all participants.

To increase the likelihood that the hand grip task would activate the explicit achievement motive, we provided participants with two sources of feedback about their performance during the task. First, exerted force was continuously displayed during the measurement period. Second, during a three-second feedback period following the measurement, the peak force that participants had exerted in the preceding measurement period was displayed together with the required force standard. This setting—together with the explicit instruction that participants’ task was to free the Ketchup bottle (see section 2.3)—should have resulted in participants perceiving the type of externally set challenge presented in verbal-symbolic format that promises potential satisfaction of the explicit achievement motive and thus activates it [8, 26, 41].

#### PRF

Participants completed a computer-based version (presented using Inquisit by Millisecond) of the achievement, affiliation, and dominance/power scales of the Personality Research Form [35] to assess their levels of sanAch, sanAff, and sanPow. Each scale included 16 descriptive statements (for example, “I will not be satisfied until I am the best in my field of work”, “I spend a lot of time visiting friends”, or “I try to control others rather than permit them to control me”) and participants indicated for each statement whether it described themselves or not. Statements from the three scales were presented alternating. sanAch constituted our main variable of interest. sanAff and sanPow were assessed to compare their impact on exerted force in the hand grip task with the impact of sanAch, which may be of interest to readers interested in the relative impact of sanAff and sanPow in this specific achievement motive-arousing situation.

### Procedure

After having provided informed consent, participants were seated at a table in front of a computer screen and introduced to the hand dynamometer that was mounted at the table in a vertical position. They learned that the device measured grip force exerted on it and were then allowed to freely explore its functionality during the next 30 seconds. During this time, the force that participants were exerting on the dynamometer was displayed on the screen, and the experimenter encouraged participants to exert varying levels of force to see how the device responded. After this familiarization period, participants performed three maximum force trials (see the maximum force trails section for details) before the experimenter gave them a sheet with instructions for the main hand grip task and left the room. For the duration of the hand grip task, participants remained alone in the room. The instruction sheet informed participants that they would be performing 60 trials of a hand grip task. They were asked to imagine that the dynamometer represented a clogged Ketchup bottle that they could free by exerting a force equal to or higher than a force standard set by the computer. All participants received the general information about the hand grip task procedure presented in the hand grip task section but did not receive any information about the specific force standards prior to or during the measurement period. Participants in the unclear task difficulty conditions (Study 1 and Study 2) learned that the force standards would vary randomly from one trial to the next one, whereas participants in the clear task difficulty condition (Study 2) were informed that the force standard would be the same in every trial. After having performed the 60 hand grip trials, participants performed again three maximum force trials in the presence of the experimenter. Participants then completed the achievement, affiliation, and dominance/power scales of Jacksons’ Personality Research Form. Finally, all participants were carefully debriefed and given an Amazon voucher worth GBP 5 for their participation.

### Data preparation and analysis

The PRF subscale items were added up to obtain participants’ sanAch, sanAff, and sanPow scores. All scales had acceptable internal consistency (Study 1: ω_t_ = .70 for sanAch, .86 for sanPow, .83 for sanAff; Study 2: ω_t_ = .71 for sanAch, .86 for sanPow, .80 for sanAff). Given that the dynamometer sampled exerted force at 10 Hz during the two-second measurement periods, 20 force values were available for each trial. The highest value of the 20 force values of a trial was used as trial peak force value and a force-time-integral (FTI) was computed by adding up all 20 force values [42]. FTIs thus indicated the total amount of effort exerted during the two-second measurement periods. However, peak force constituted our primary measure given that it was instrumental for success (exerted peak force was compared with the force standard to determine success in a trial).

The highest peak force value during the maximum force trials was used as a participant’s maximum force score. In Study 1, this was based on all six maximum force trials but in Study 2, we only used the three post-task maximum force trials to determine maximum force scores given that a programming error prevented our software from saving exerted force during the pre-task maximum force trials in the clear-difficulty condition. For each participant, we computed a task peak force score as arithmetic average of all 60 peak force values.

In both studies, we tested our predictions about the impact of sanAch on exerted force under conditions of unclear task difficulty by calculating Pearson correlation coefficients between peak force scores and sanAch. In Study 2, this analysis only considered participants in the unclear-task difficulty condition. We also tested the relative importance of sanAch as predictor of exerted force by comparing the Pearson correlation coefficients between the three motive scores (achievement, affiliation, and power) and peak force scores using Dunn and Clark’s *z* [43] and by calculating Bayes Factors comparing regression models that tested for a positive association between the individual motive scores and peak force scores [44, 45]. In Study 2, we additionally compared the difference between sanAch impact under conditions of unclear and clear difficulty by comparing the Pearson correlation coefficients between sanAch and peak force scores between both difficulty conditions using Fisher’s *z* and by calculating Bayes Factors that compared the fit of a regression model that only included sanAch as predictor with a model that included the interaction of sanAch with difficulty as predictor. All analyses were conducted using R [46] and the psych [47], cocor [48], and BICpack [49] packages. *P*-value based tests that addressed the following a-priori directional hypotheses were conducted one-tailed: 1) the postulated positive relationship between sanAch strength and exerted force under conditions of unclear task difficulty, 2) the prediction that sanAch would be more positively associated with exerted force than sanAff or sanPow under conditions of unclear task difficulty, and 3) the hypothesis that the sanAch-exerted-force relationship would be more positive under conditions of unclear task difficulty than under conditions of fixed task difficulty. All other *p*-value based tests were conducted two-tailed. The strength of evidence provided by the observed Bayes Factors was interpreted according to Andraszewicz et al. [50] modification of Jeffreys’ classification scheme.

## Results

### Study 1

#### Maximum force trials

Participants’ mean maximum force was 230.76 N (*SD* = 68.69; pre-task *M* = 222.01 N, *SD* = 72.74, range = 85.08–467.40; post-task *M* = 212.60 N, *SD* = 69.73, range = 98.10–476.12). There was no evidence that exerted maximum force differed before and after the task, *t*(39) = 1.84, *p* = .07, *d* = 0.41, 95% CI [-0.03, 0.85]. Participants’ maximum force was significantly correlated with task peak force, *r*(38) = .77, *p* < .001, 95% CI [.61, .87], and task FTI, *r*(38) = .65, *p* < .001, 95% CI [.43, .80]. Moreover, maximum force was significantly correlated with sanAff, *r*(38) = -.58, *p* < .001, 95% CI [-.76, -.33], but not with sanAch, *r*(38) = .12, *p* = .45, 95% CI [-.20, .42], or sanPow, *r*(38) = -.16, *p* = .31, 95% CI [-.45, .15]. Given the significant relationship between force exerted during the main task and participants’ maximum force and given that it is common to use relative force values in hand grip studies [51–53], we decided to analyze relative peak force values expressed in percentages of maximum force.

#### Relationship between exerted force and motive scores

**Fig 2:**
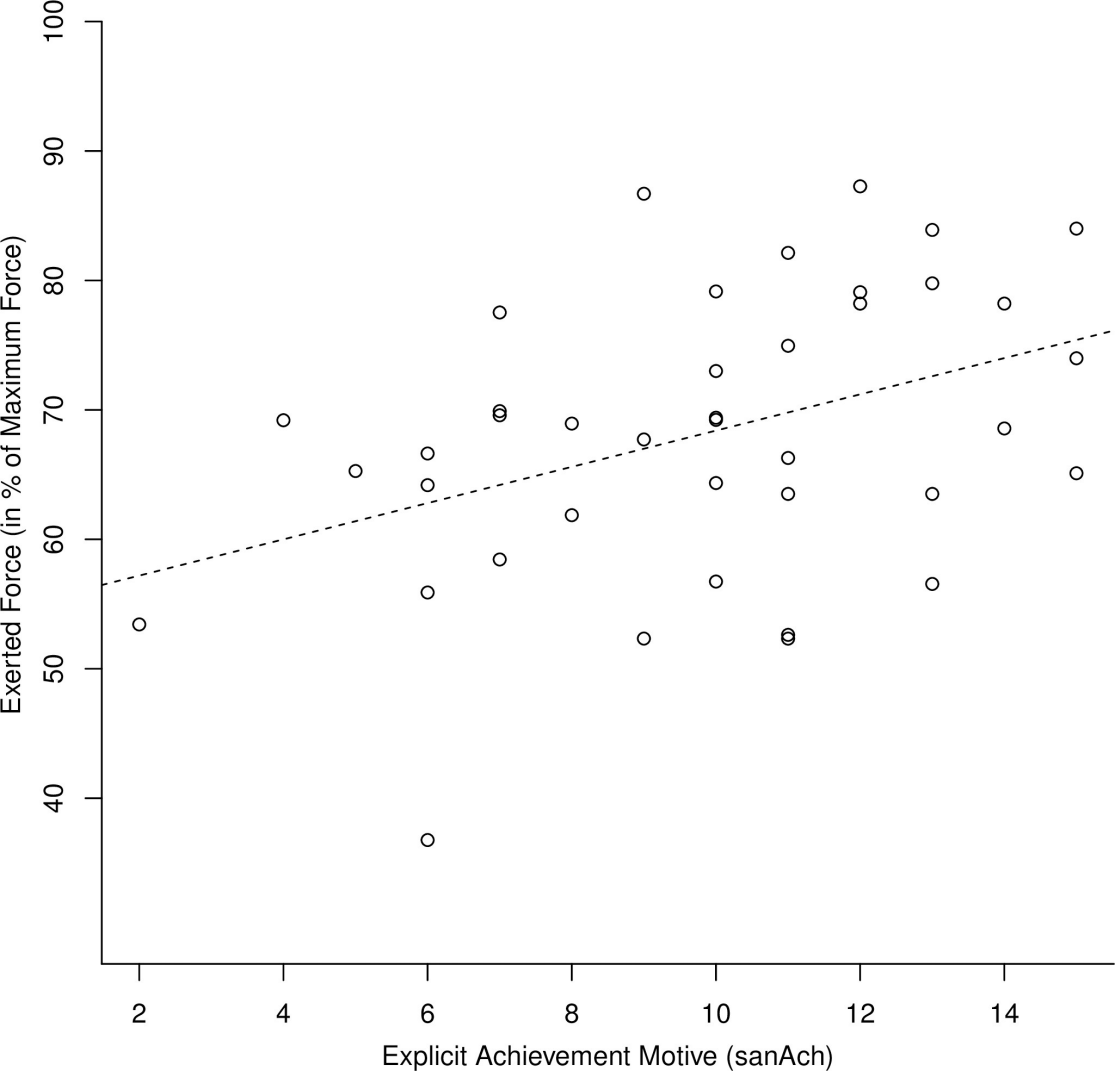
Relationship between explicit achievement motive strength and relative exerted force in Study 1. The dashed line shows the best fit regression line.

**Fig 3:**
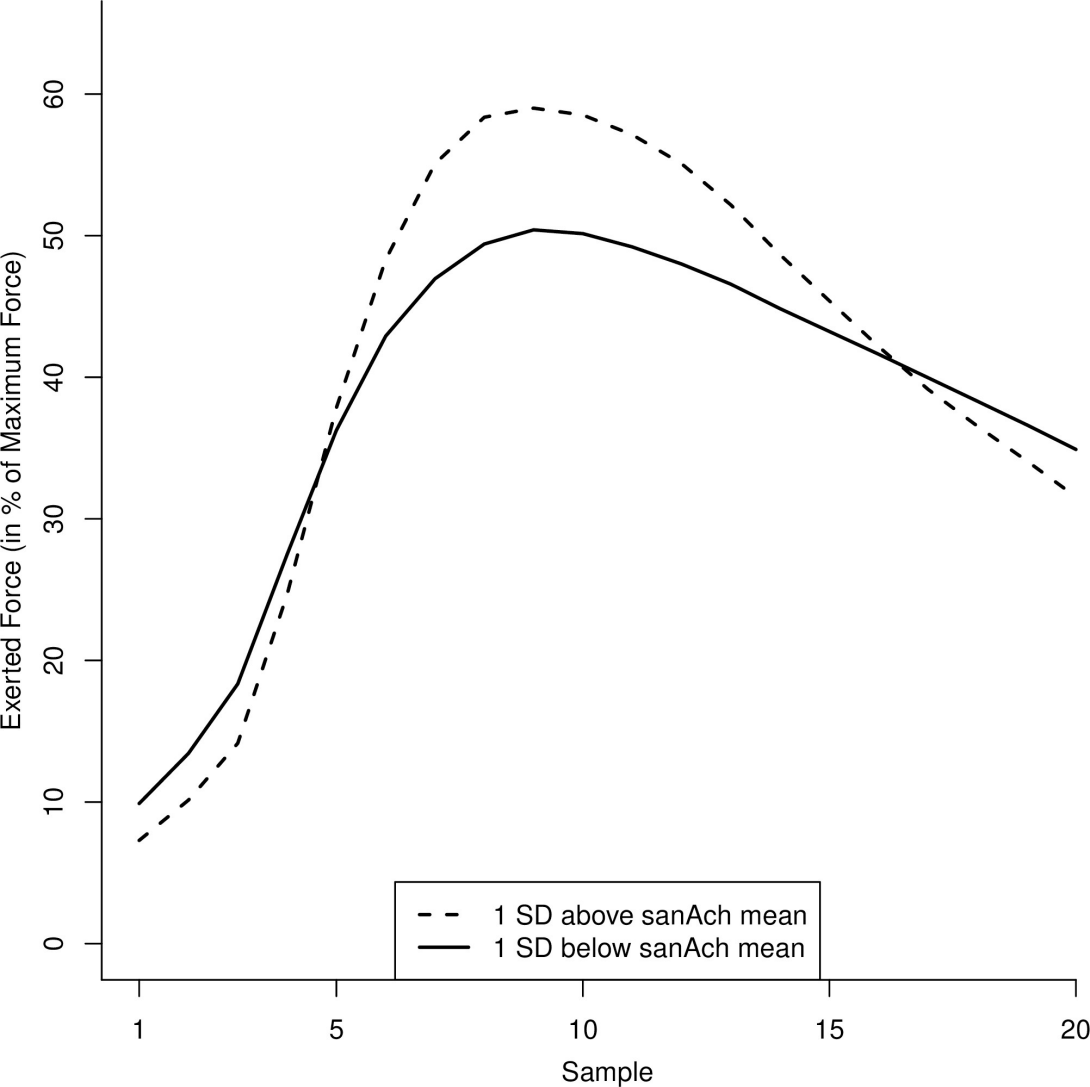
Evolution of relative exerted force during a trial as a function of explicit achievement motive strength in Study 1. The graph is based on predicted values generated from regression equations for individuals one SD above and below the mean explicit achievement motive (sanAch) strength. The x-axis refers to the 20 force samples collected during each task trial.

Means and standard deviations of force values and motive scores as well as the correlation coefficients between these variables can be found in Table 1. As hypothesized, participants’ sanAch was positively associated with relative peak force (*p* = .005): The higher a participant’s sanAch, the higher the relative force that she/he exerted in the hand grip task. Fig 2 illustrates this relationship and Fig 3 presents the evolution of exerted force across the 2-second measurement period as a function of sanAch. Participants’ sanAch was also positively correlated with absolute force (*p* = .01), but there was no evidence for a positive relationship between sanAch and FTI (*p* = .08).

**Table 1:** Descriptive statistics and Pearson correlation coefficients for force values and motive scores in Study 1.

Variable	*M*	*SD*	1	2	3	4	5	6
1. Relative peak force ^a^	68.16	11.06	—					
2. Absolute peak force ^b^	156.77	52.20	.54 [.28, .73]	—				
3. FTI ^b^	1809.35	694.38	.28 [-.03, .55]	.75 [.58, .86]	—			
4. sanAch	9.82	3.19	.40 [.16, 1.00]	.36 [.10, 1.00]	.23 [-.03, 1.00]	—		
5. sanPow	8.30	3.97	.14 [-.18, .43]	-.04 [-.35, .28]	-.01 [-.32, .30]	.33 [.03, .59]	—	
6. sanAff	10.62	3.77	.03 [-.28, .34]	-.43 [-.65, -.14]	-.49 [-.69, -.21]	.05 [-.27, .35]	.26 [-.06, .53]	—

To prevent type-I error inflation, *p*-value based tests were only conducted for the correlation coefficients central to our hypotheses. The associated *p*-values are presented in the text. 95% confidence interval limits are presented in square brackets.

^a^ Relative peak force is expressed in percentage of maximum force.

^b^ Absolute peak force and FTI are in Newton.

Demonstrating the strong relative impact of sanAch as predictor of exerted force in the hand grip task, the correlation coefficient between sanAch and relative force was significantly more positive than the correlation coefficient between sanAff and relative force, *z* = 1.71, *p* = .04. However, even if the correlation coefficient between sanAch and relative force was more positive in absolute terms than the correlation coefficient between sanPow and relative force, this difference was not significant, *z* = 1.46, *p* = .07. The Bayes Factors provided strong evidence in favor of the sanAch model in comparison with the sanPow (*BF* = 28.50) and sanAff (*BF* = 58.03) models demonstrating that sanAch was more predictive of relative peak force than sanPow or sanAff.

### Study 2

#### Maximum force trials

Participants’ mean maximum force scores were 227.86 N (*SD* = 69.66, range = 136.37–438.39) in the unclear-difficulty condition and 201.37 N (*SD* = 72.37, range = 83.32–322.68) in the clear-difficulty condition. There was no evidence that exerted maximum force differed between the two difficulty standard conditions, *t*(38) = 1.18, *p* = .25, *d* = 0.37, 95% CI [-0.25, 1.00]. Maximum force scores were significantly correlated with task peak force scores, *r*(38) = .73, *p* < .001, 95% CI [.55, .85], and task FTI, *r*(38) = .37, *p* = .02, 95% CI [.07, .61]. They were also significantly associated with sanPow, *r*(38) = .34, *p* = .03, 95% CI [.03, .59], but not with sanAch, *r*(38) = -.07, *p* = .67, 95% CI [-.37, .25], or sanAff, *r*(38) = -.03, *p* = .83, 95% CI [-.34, .28]. Given the relationship between participants’ maximum force and the force exerted during the main task as well as to be consistent with Study 1, we decided to focus our analysis again on relative peak force scores expressed in percentages of maximum force.

#### Relationship between exerted force and motive scores

**Fig 4:**
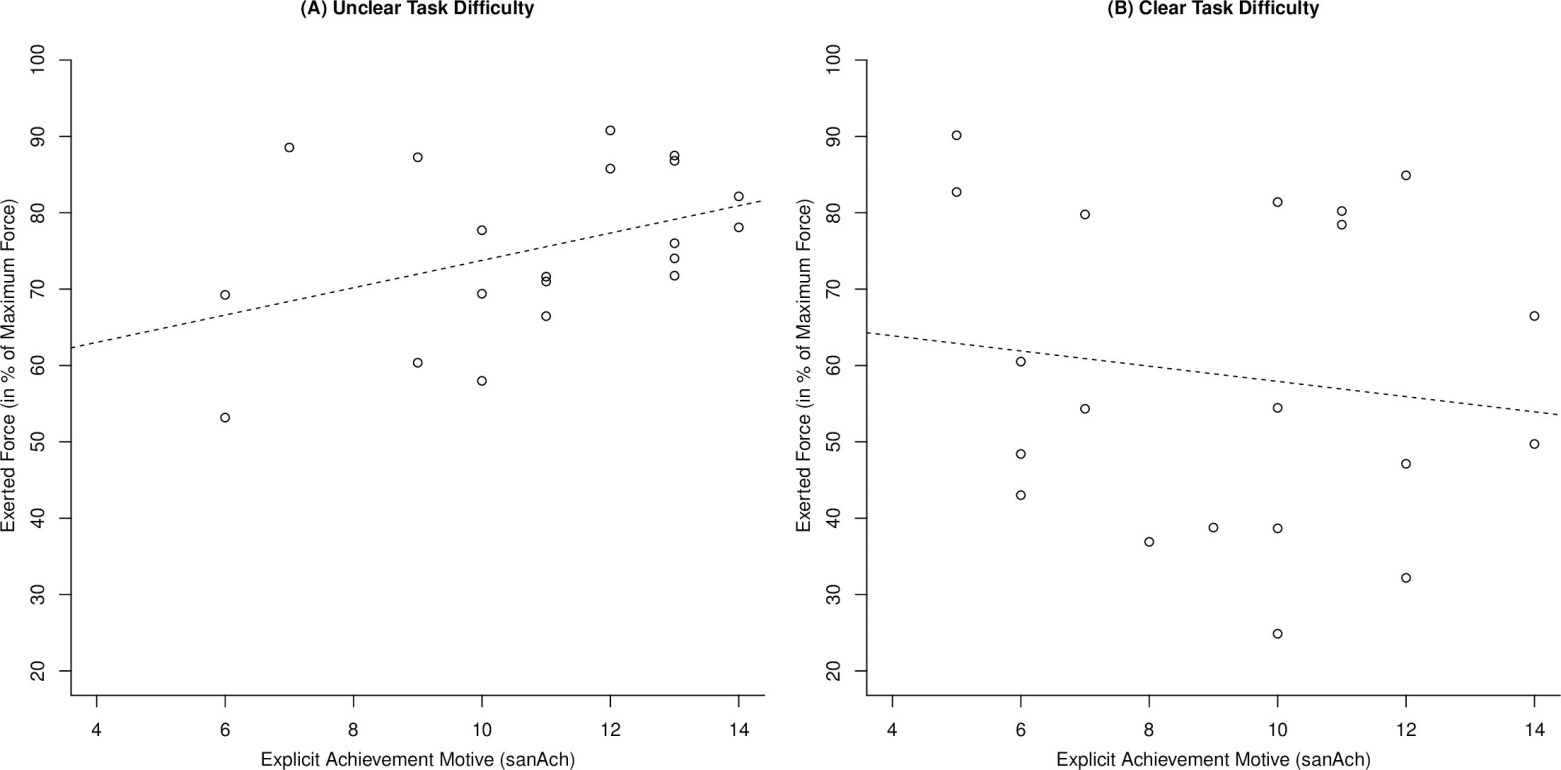
Relationship between explicit achievement motive strength and relative exerted force in Study 2. Dashed lines are best fit regression lines.

**Fig 5:**
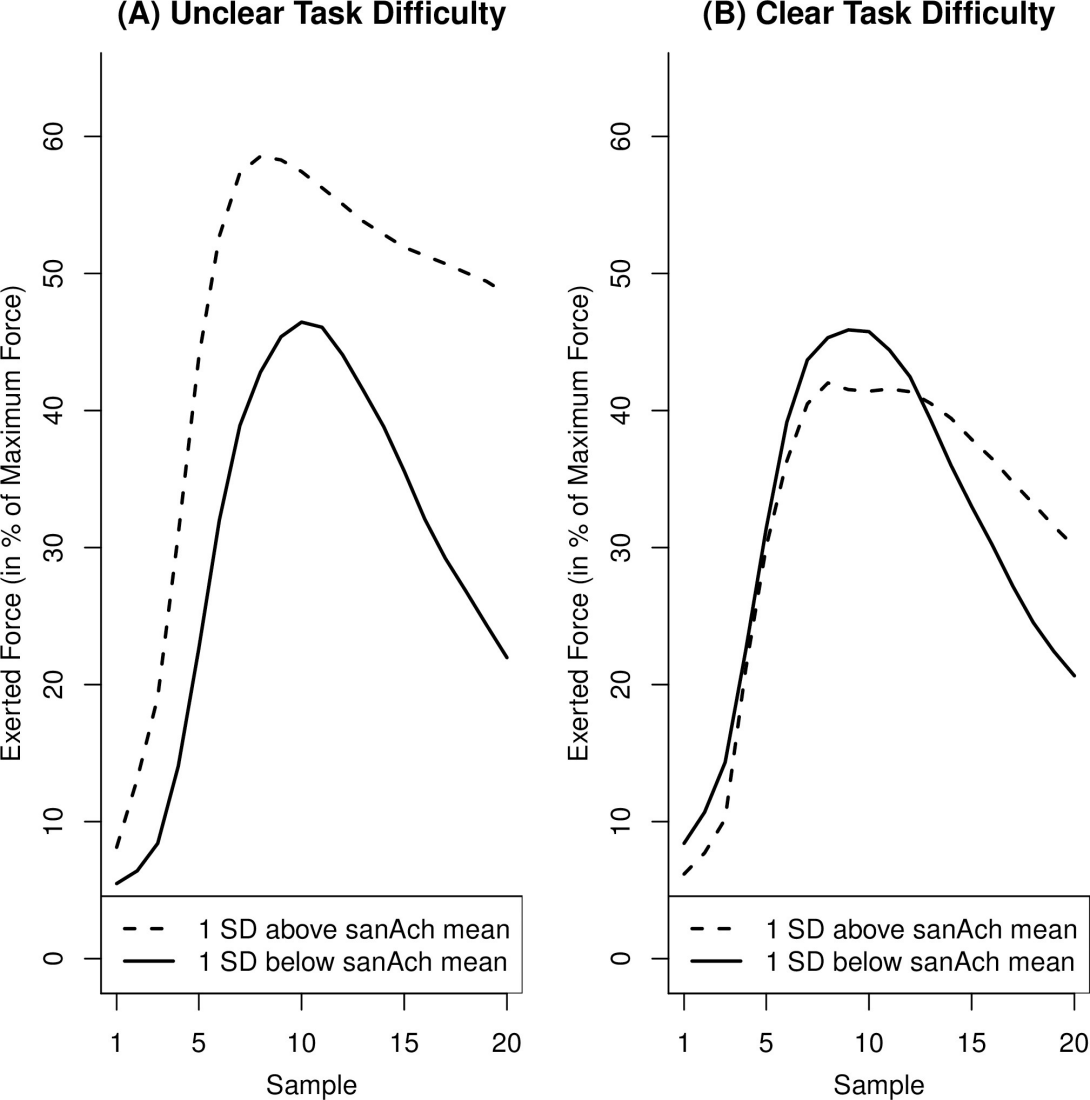
Evolution of relative exerted force during a trial as a function of explicit achievement motive strength in Study 2. The graphs are based on predicted values generated from regression equations for individuals one SD above and below the mean explicit achievement motive (sanAch) strength. The x-axis refers to the 20 force samples collected during each task trial.

Table 2 displays means, standard deviations, and bivariate correlations of force and motive scores. As predicted, sanAch was positively correlated with relative peak force in the unclear-difficulty condition (*p* = .04) but not in the clear-difficulty condition (*p* = .72). There was no evidence for positive relationships between sanAch and absolute peak force or FTI in the unclear-difficulty (*p*s > .13) or clear-difficulty conditions (*p*s > .31). Fig 4 illustrates the relationship between sanAch and relative peak force in the two difficulty conditions and Fig 5 shows the evolution of exerted force across the 2-second measurement period as a function of sanAch and difficulty condition.

**Table 2:** Descriptive statistics and Pearson correlation coefficients for force and motive scores in Study 2.

Variable	*M*	*SD*	1	2	3	4	5	6
Unclear difficulty								
1. Relative peak force ^a^	75.29	10.81	—					
2. Absolute peak force ^b^	172.26	63.21	.48 [.05, .76]	—				
3. FTI ^b^	1923.78	653.70	.27 [-.20, .63]	.49 [.06, .77]	—			
4. sanAch	10.85	2.48	.41 ^c^ [.04, 1.00]	-.22 [-.56, 1.00]	.26 [-.14, 1.00]	—		
5. sanPow	7.95	4.27	-.24 [-.61, .23]	.03 [-.41, .47]	.18 [-.28, .58]	-.19 [-.59, .27]	—	
6. sanAff	10.30	3.96	.25 [-.21, .62]	.14 [-.31, .55]	-.13 [-.54, .33]	.04 [-.41, .48]	.37 [-.09, .70]	—
Clear difficulty								
1. Relative peak force ^a^	58.66	20.33	—					
2. Absolute peak force ^b^	114.01	53.12	.58 [.18, .81]	—				
3. FTI ^b^	1168.78	768.62	.77 [.50, .91]	.86 [.66, .94]	—			
4. sanAch	9.25	2.86	-.14 ^c^ [-.49, 1.00]	.02 [-.36, 1.00]	.12 [-.27, 1.00]	—		
5. sanPow	6.05	3.91	-.02 [-.46, .42]	.42 [-.03, .72]	.31 [-.15, .66]	.32 [-.14, .67]	—	
6. sanAff	10.45	3.09	.14 [-.33, .55]	.07 [-.38, .50]	.20 [-.27, .59]	.06 [-.39, .49]	-.10 [-.52, .36]	—

To prevent type-I error inflation, *p*-value based tests were only conducted for correlation coefficients central to our hypotheses. The associated *p*-values are presented in the text. 95% confidence interval limits are presented in square brackets.

^a^ Relative peak force is expressed in percentage of maximum force.

^b^ Absolute peak force and FTI are in Newton.

^c^ Controlling for gender did virtually not change the correlation coefficients between sanAch and relative peak force.

A comparison of the correlation coefficients of sanAch with relative peak force scores between the unclear-difficulty and the clear-difficulty conditions was significant, *z* = 1.68, *p* = .05, supporting our hypothesis that the relationship between sanAch and exerted force would be more positive under conditions of unclear difficulty than under conditions of clear difficulty. Moreover, the Bayes Factors provided strong evidence in favor of the regression model including the sanAch-difficulty interaction as predictor and against the model including only sanAch as predictor (*BF* = 28.91).

Further demonstrating the relative impact of sanAch as predictor of exerted force under conditions of unclear task difficulty, the correlation coefficient between sanAch and relative peak force in the unclear-difficulty condition was significantly more positive than the correlation coefficient between sanPow and relative peak force in that condition, *z* = 1.80, *p* = .04. However, the correlation coefficients of sanAch and sanAff with relative peak force in the unclear-difficulty condition did not differ from one another, *z* = 0.52, *p* = .30. Bayes Factors also provided moderate to strong evidence in favor of the regression model including sanAch as predictor of relative peak force under conditions of unclear difficulty and against the models using sanPow (*BF* = 22.91) and sanAff (*BF* = 3.70) as predictors.

## Discussion

In both studies, we found the predicted positive relationship between the strength of the explicit achievement motive and effort under conditions of unclear, unpredictable task difficulty: the higher the strength of the achievement motive, the higher the force that participants exerted. Study 2 replicated the positive relationship found in Study 1 and revealed that this relationship was specific to tasks with unclear difficulty. If the difficulty of the task was clear, the strength of the explicit achievement motive did not predict exerted force. Replicating preceding work [54–56], we also observed that the explicit achievement motive was more predictive of effort investment than the explicit power or affiliation motives. As expected, the employed hand grip task constituted an achievement-related context that activated the explicit achievement motive more than the power or affiliation motives.

Our results may seem obvious from a motivational intensity theory perspective but it should be noted that the achievement motive literature has not yet fully adopted the idea that aroused motives do not always lead to increased effort, persistence, or performance. A large part of the motive research is still based on the assumption that motives exert a direct impact on behavior once that they have been activated by an opportunity for motive satisfaction [9, 57, 58]. There are a few exceptions that provided more sophisticated models on how motives interact with situational characteristics to influence different aspects of behavior [25–27, 59] but many publications have an explanatory gap between the aroused motive and the behavioral consequences. For instance, it often remains unclear how a high motive strength should lead to increased performance. Assuming that high motive strength leads to increased effort and persistence, which in turn increases performance, does not fully close the explanatory gap given that effort and persistence are only two of several determinants of performance [60, 61]. Even if our work does not address performance effects in the terms of success and failure, it challenges the idea that motive activation is sufficient for motive effects on behavior. It clearly demonstrates that an activated explicit achievement motive only has a direct impact on the effort invested in instrumental behavior if the difficulty of the behavior is unclear or unpredictable. If the difficulty of the behavior is well-known and fixed to a certain difficulty standard (i.e., there is a known and clear criterion that determines success and failure), an activated explicit achievement motive does not directly affect effort investment.

It might seem surprising that we predicted and found effects of the explicit achievement motive on effort given that the energetic and performance-related aspects of behavior have often been associated with the implicit motive, not the explicit motive [10, 26, 62]. However, a closer examination of the behavior that we assessed—exerting force in the Ketchup hand grip task—reveals that it has many features that have been attributed to behavior that should be influenced by the explicit achievement motive [25, 27]. For instance, exerting force in the hand grip task constitutes certainly not a spontaneous behavior but a deliberate decision to exert force. Moreover, it is more respondent behavior than operant behavior given that there were specific stimuli eliciting the response (for instance, the instruction to squeeze the dynamometer to free the Ketchup bottle). Exerted force also represents a declarative measure of motivation given that exerting force in the hand grip task is a voluntary, consciously controlled process. In sum, even if exerted force might look at first sight to be a behavioral response for which implicit motive strength should be predictive, a closer look reveals that exerting force in the Ketchup hand grip tasks constitutes the type of deliberate, controlled, and respondent behavior for which explicit motive strength should be relevant.

Our work differs from a large part of the preceding work on motivational intensity theory [14] regarding the employed effort measure. Instead of using sympathetic-driven cardiovascular measures to test our effort-related predictions, we assessed the force exerted in an isometric hand grip task. Using exerted force in the presented work had a number of advantages. First, a lot of the current work on the achievement motive examines its impact on physical, mainly sports-related tasks [9, 33, 34]. Assessing physical effort in a motor task is obviously more relevant to this work than cardiovascular responses associated with mental effort. Second, it only requires a couple of seconds to measure the force response in one trial. Consequently, many trials can be presented in a task to collect a large number of effort measurements. Given that each individual measurement is affected by random noise resulting from factors not related to the main manipulations (e.g., the participant being distracted by a noise outside the lab and missing the start of the measurement period), a larger number of effort measurements makes it more likely that the average of the individual measurements accurately captures the effect of the main manipulations on effort. Third, exerted force provides a good indication of the relative amount of energy used to execute the hand grip task and provides thus a valid measure to test motivational intensity theory’s predictions related to energy investment (i.e., effort). Given the close connection between exerted force in isometric tasks and the amount of consumed adenosine triphosphate—the primary energy for most energy requiring actions of the human body—exerted force enables specific tests of the determinants of the amount of energetic resources that are invested in instrumental tasks [37]. Independent of the differences with the cardiovascular measures employed in most of the research on motivational intensity theory, our findings fit well with preceding work on the theory. Like other researchers who found that variables related to success importance exert a direct impact on effort under conditions of unclear and unfixed task difficulty but not under conditions of fixed task difficulty [14, 63, 64], we observed that the impact of the explicit achievement motive was moderated by clarity of task difficulty.

A critical reader might wonder about the implications of the confound between clarity of difficulty and task difficulty in Study 2. Given that we presented a broad range of force standards (including five standards that exceeded participants’ maximum force) in the unclear condition, the average difficulty in this condition was higher than in the clear condition where we only presented low-difficulty trials with a force standard of 60 Newton. This confound does, however, not challenge our interpretation in terms of clarity of difficulty given that an interpretation in terms of fixed and clear task difficulty fails to explain the observed relationship between achievement motive strength and exerted force. If participants had perceived the unclear difficulty task as one with a clear and high difficulty, their exerted force should not have increased in proportion to their achievement motive strength. According to motivational intensity theory, task difficulty directly determines effort if participants have a clear idea about task difficulty. Achievement motive strength, as a variable that influences success importance, should not have a direct impact. Correspondingly, exerted force should either have been high for all participants independent of their achievement motive strength or shown a twofold pattern with some participants exerting a high force and others disengaging. Fig 4 clearly shows that this was not the case. The only theory-based interpretation that can explain the finding that achievement motive strength was associated with exerted force in one condition but not in the other is thus one in terms of clarity of difficulty.

The quasi-experimental design of our studies obviously limits the conclusions that can be drawn from the presented data. Our findings cannot provide strong evidence for the causal link between the explicit achievement motive and effort suggested by our theoretical analysis. It is possible that the achievement motive was not influencing effort investment but that the effort that participants had invested in the task determined the achievement motive strength that they reported. It is also not excluded that the observed positive relationship between achievement motive strength and effort was due to a third variable that affected both the explicit achievement motive and exerted force in the same manner. One way to improve the design and provide more direct evidence for the causal effect of the achievement motive on effort might be to manipulate the situational characteristics so that the achievement motive is either activated or not. However, it is important to note that the impact of clarity of difficulty that we observed in Study 2 is not vulnerable to the quasi-experimental design critique. The observed relationships between the achievement motive and effort may not provide strong evidence for the causal effect of the achievement motive on effort but the manipulation of clarity of difficulty demonstrates that variations in the clarity of task difficulty cause changes in the relationship between achievement motive strength and effort.

In this article, we presented a theoretical analysis of achievement motive effects on effort investment in instrumental tasks and provided empirical evidence for some of the predictions of this analysis. We demonstrated that the impact of the explicit achievement motive on effort depends on the clarity of task difficulty. If the difficulty of a task was known and fixed, achievement motive strength did not predict effort. However, if the difficulty standard was unknown and unpredictable to participants, achievement motive strength predicted effort. Task characteristics—in particular, the predictability of task difficulty—are thus important factors to consider when using achievement motive strength to predict engagement in various achievement-related tasks.
